# Specific and intrinsic sequence patterns extracted by deep learning from intra-protein binding and non-binding peptide fragments

**DOI:** 10.1038/s41598-017-14877-w

**Published:** 2017-11-02

**Authors:** Yuhong Wang, Junzhou Huang, Wei Li, Sheng Wang, Chuanfan Ding

**Affiliations:** 1 Department of chemistry and Laser Chemistry Institute, Fudan University, Shanghai, 200433 P.R. China; 2 Department of Computer Science and Engineering, The University of Texas at Arlington, Arlington, TX 76019 USA; 3 School of life science, Jilin University, Changchun, 130012 P.R. China

## Abstract

The key finding in the DNA double helix model is the specific pairing or binding between nucleotides A-T and C-G, and the pairing rules are the molecule basis of genetic code. Unfortunately, no such rules have been discovered for proteins. Here we show that intrinsic sequence patterns between intra-protein binding peptide fragments exist, they can be extracted using a deep learning algorithm, and they bear an interesting semblance to the DNA double helix model. The intra-protein binding peptide fragments have specific and intrinsic sequence patterns, distinct from non-binding peptide fragments, and multi-millions of binding and non-binding peptide fragments from currently available protein X-ray structures are classified with an accuracy of up to 93%. The specific binding between short peptide fragments may provide an important driving force for protein folding and protein-protein interaction, two open and fundamental problems in molecular biology, and it may have significant potential in design, discovery, and development of peptide, protein, and antibody drugs.

## Introduction

Protein folding and protein-protein interaction are two fundamental, long-standing problems in molecular biology, and their importance can hardly be overestimated. The protein folding problem is to predict three dimension structure (3D) of a protein from its amino acid sequence (1D)^1^. The protein-protein interaction (PPI) is to predict specific binding/interaction between two or more proteins^2^. Life depends upon its components, these components’ functioning, and information flow between them. Protein is one fundamental component of life, and its function depends upon 3D structure. PPI is the molecule basis of information flow.

Experimental approaches for determination of protein structure and PPI have advanced at an ever-faster rate^2,3^, but they remain expensive, time-consuming, and insufficient. For example, it is difficult to detect weak, but biological important interactions between proteins. While computational approaches are fast and inexpensive, their current roles remain supplementary. It remains a highly challenging task to predict protein structures and PPI *de novo* ^2,3^ despite the huge advances in computing power.

For protein folding process, three main models have been proposed^4^. The first assumes a bottom up, sequential, and stepwise formation process^5^. Secondary structure elements are formed first, followed by their diffusion, collision and coalescence to form tertiary structure. The second is similar to the first, but it assumes nucleation^6^ first, followed by propagation of structures. The third, more modern one, assumes that the initial steps involve hydrophobic collapse^7–10^, followed by formation of secondary structure elements and correct packing inside a relatively compact volume.

Theoretically protein folding and PPI are mainly driven by non-covalent, weak interactions^11,12^. van der Waals interactions, the most common one, are short range forces and occur when atoms come close to each other. Hydrophobic interactions and hydrogen bonds both make large contributions to protein stability. The burial of nonpolar side chains removes them from water, enhances van der Waals interaction, and leads to tight packing in the protein interior. The hydrogen bonds take place between a proton donor and a proton acceptor. Electrostatic interactions, unlike van der Waals forces and hydrogen bonds, are long range ones; they remain relevant beyond the limits of the closest neighbors.

The limited applications of computational approaches in prediction of protein structure and PPI suggest a need for novel ideas, in particular for force fields. This study is one such effort, and it started from our earlier interests in binding or spatially close peptide fragments in globular proteins^13^. Computational approaches for protein folding and PPI problem starts from the assumption that a protein’s native conformation corresponds to its global free energy minimum^1^ and binding peptide fragments are brought together after 3D structures are formed. However, we did observe interesting patterns between intra-protein binding peptide fragments. Thus, we proposed an alternative mechanism: binding peptide fragments are formed first and drive the formation of protein 3D structure and PPI. Unfortunately, available protein structure data in early 1990s was not sufficient for further exploration.

## Results

In this study, we examined this alternative hypothesis, and our main thinking is that if this hypothesis is true, binding peptide fragments must have specific and intrinsic sequence pattern that are distinct from non-binding ones. If sufficient number of samples is collected, binary classification algorithm in machine learning can be applied to identify such intrinsic patterns and distinguish binding from non-binding samples.

**Figure 1:**
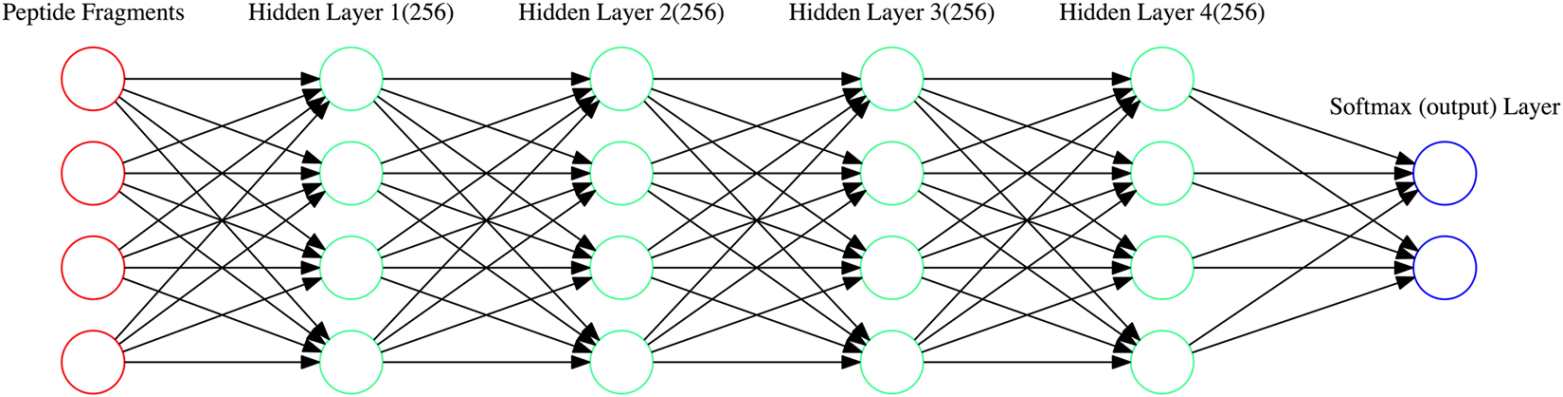
Illustration of the forward deep learning model for classification of binding and non-binding peptide fragments.

We used Deep Learning^14^ methods for this classification. Comparing with traditional machine learning method, Deep Learning methods allow deep neural networks discovering complex relationship between input variables and output observations and are more efficient for problems of large sample size. The deep neural network we used contains an input layer, four hidden layers, and an output layer (Fig. 1). In this study, the input variables are the amino acid sequences of peptide fragments, and the output is a binary classification: binding or non-binding. Each layer consists of a number of neurons or nodes. A typical machine learning process consists of two steps: training and testing. Training is performed on training data set. In this step, the connection weights between neurons are iteratively adjusted so the generated output values are as close to the expected ones as possible. In the testing step, the trained model is applied to test data set, which is distinct from the training data set, and collect a number of benchmarks. One common benchmark is the accuracy or the percentage of the samples in test data set that have been correctly predicted.

We focused upon specific interactions between two and three short peptide fragments, named as peptide triad (PT) and duo (PD), respectively, after common music terms. A binding peptide triad (BPT) and duo (BPD) and a non-binding peptide triad (NBPT) and duo (NBPD) are defined as PT and PD having all pair-wise minimum distances between center residues <5.0 Å and >30 Å, respectively. For fragments having even number of residues, the minimum distance between two fragments is calculated as the average of minimum distances between two center residues. The minimum distance between two residues is defined as the minimum distance between all non-hydrogen atoms of the two residues. Binding and non-binding peptides are solely defined using distance cutoff.

From 12,946 X-ray protein structures^15^, we extracted 1.2–3.5 millions of BPTs, 1.4–4.5 millions of NBPTs, and 0.4–0.9 millions of BPD and NBPDs (Table 1). We designed a neural network (Fig. 1) and performed supervised deep learning classification algorithm on the combined 2.6–8.0 and 0.8–1.9 million of PT and PD samples, respectively. The input is the amino acid sequences of peptide triads or duos. Each hidden layer consists of 256 nodes. The output layer has two nodes for binding or non-binding.

**Table 1:** Classification results of binding and non-binding peptide fragments on test data sets in terms of accuracy, area under the ROC curve (AUC-ROC), F-Score, precision, and recall.

Peptide fragments	No of BPFS^1^	No of NBPFS^2^	Final loss	Accuracy	AUC-ROC	F-Score	Precision	Recall
**Peptide Triads**								
3 × 2	3,506,094	4,573,534	26,417	0.739	0.807	0.675	0.735	0.624
3 × 3	3,202,563	3,727,467	19,058	0.793	0.871	0.768	0.797	0.742
3 × 4	2,454,016	2,821,820	11,256	0.849	0.924	0.834	0.852	0.817
3 × 5	1,943,073	2,346,432	5,491	0.915	0.969	0.906	0.911	0.902
3 × 7	1,561,153	1,744,130	3,450	0.931	0.979	0.927	0.938	0.917
3 × 9	1,276,502	1,398,539	2,815	0.923	0.975	0.919	0.915	0.923
**Peptide Duos**								
2 × 3	938,992	972,945	7,463	0.620	0.668	0.577	0.639	0.526
2 × 5	692,955	658,592	2,689	0.841	0.911	0.841	0.861	0.822
2 × 7	526,614	506,836	1,572	0.836	0.905	0.835	0.856	0.816
2 × 9	419,945	420,781	1,238	0.770	0.845	0.758	0.800	0.721

Loss function is optimized using the ADAM optimizer and a mini-batch size is 128. Other optimized parameters are given in Table 2. ^1^Number of binding peptide fragments samples, and ^2^number of non-binding fragments samples.

The combined samples are randomly split into three data sets: 80% for training, 10% for validation, and 10% for test. The neural network was trained by minimizing the “cross-entropy” loss function using the ADAM optimizer^16^, a mini-batch size of 128, and other optimized parameters (Table 2). The training process was monitored by checking accuracy of the validation data set, and terminated when no further improvement was observed. The trained models were applied to the test data set for benchmarking in terms of accuracy, area under the ROC curve (AUC-ROC), F-Score, precision and recall. For purpose of negative control, the labels (binding or non-binding) of samples were randomized, and the same training procedure and benchmarking were performed.

**Table 2:** The regularization coefficient and starting learning rate for the neural network training; both were optimized after a grid search.

Peptide fragments	Regularization coefficient	Starting learning rate
3 × 2	0.0000010	0.0006
3 × 3	0.0000010	0.0006
3 × 4	0.0000010	0.0006
3 × 5	0.0000010	0.0006
3 × 7	0.0000025	0.0006
3 × 9	0.0000025	0.0008
2 × 3	0.0000010	0.0008
2 × 5	0.0000010	0.0008
2 × 7	0.0000010	0.0008
2 × 9	0.0000010	0.0008

**Figure 2:**
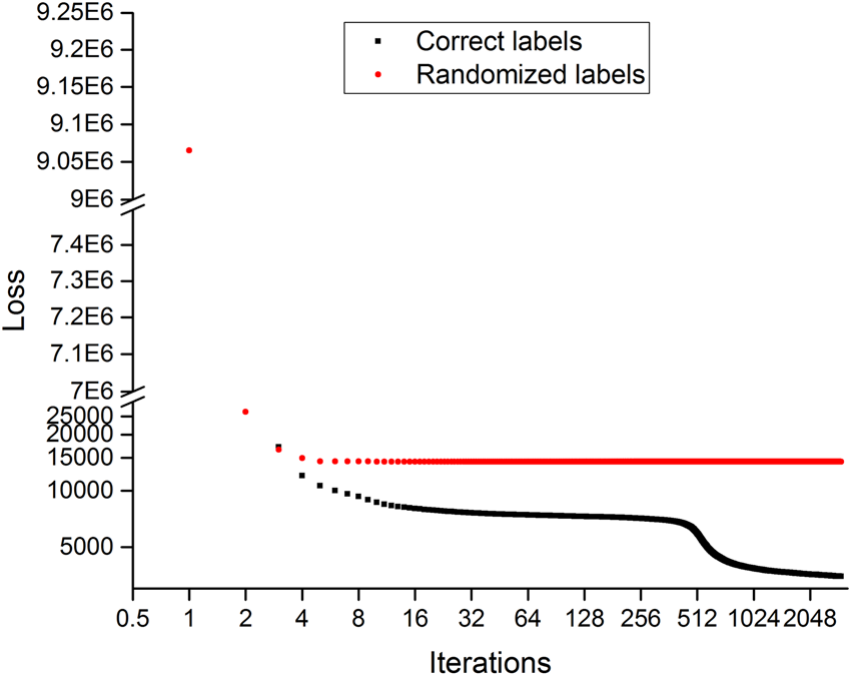
Model loss on training data sets for peptide triads of 7 residues with correct and randomized labels. Sample size of the training data set is 2,644,109.

**Figure 3:**
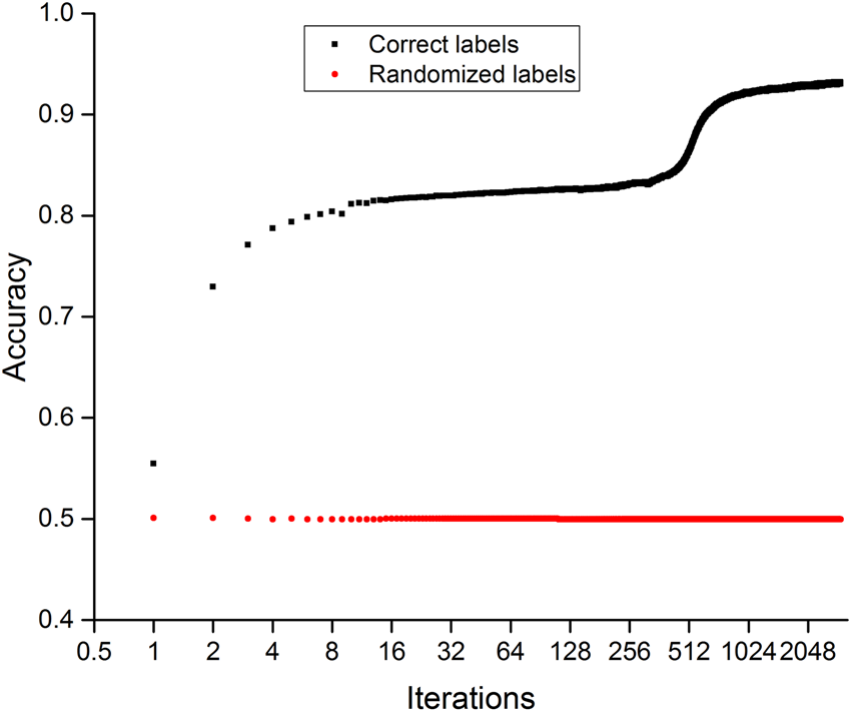
Model accuracy on validation data sets for peptide triads of 7 residues with correct and randomized labels. Sample size of the validation data set is 330,869.

For PTs of 7 residues, the loss function of training data set dropped fast in the first 10 iterations, followed by noticeably slower decrease (Fig. 2). The prediction accuracy on validation data set increased fast in the first 10 iterations, followed by remarkably slower improvement (Fig. 3). For PTs of 7 residues of randomized labels, the loss function decreased fast in the first a few iterations and then stayed constant; the accuracy stayed at 0.5 throughout the training process. PTs of 2, 3, 4, 5, and 9 residues and PDs of 3, 5, 7, and 9 residues had very similar profiles.

**Figure 4:**
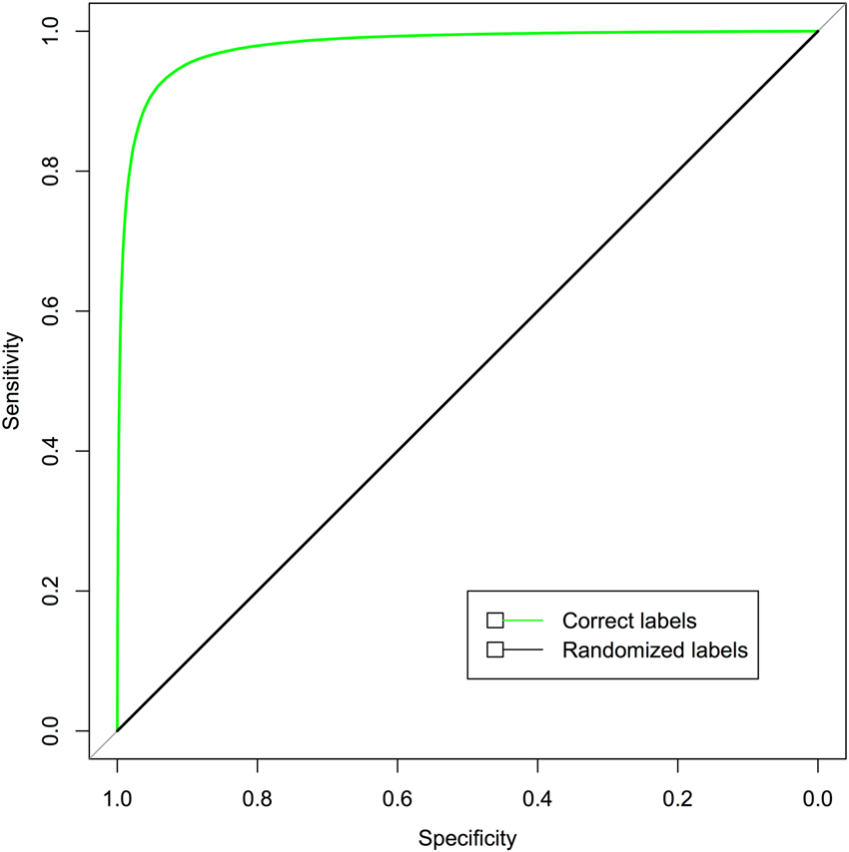
ROC curves for binding peptide triads of 7 residues. Total sample size of test data is 330,305. The AUC-ROC are 0.979 and 0.500 for test data of correct and randomized labels, respectively.

The trained models were applied into test data sets and the performance benchmarks are listed in Table 1. For PTs of 2–9 residues, the accuracy increases from 0.74, 0.79, 0.84, 0.912, to 0.931 and then comes down to 0.923. PTs of 7 residues have the best accuracy of 0.931 and AUC-ROC of 0.979 (Fig. 4), and this finding seems to be consistent with recent screening results^17^. PDs of 3–9 residues have the accuracy of 0.620, 0.841, 0.836, and 0.770. PDs of 5 residues have best accuracy of 0.841 and AUC-ROC of 0.911. No meaningful models could be learned from PTs and PDs of randomized labels (Table 3), and the AUC-ROC have perfectly random values of 0.5.

**Table 3:** Classification results of binding and non-binding peptide fragments with randomized labels on test data sets in terms of accuracy, area under the ROC curve (AUC-ROC), F-Score, precision, and recall.

Peptide Fragments	No of BFSs^1^	No of NBFSs^2^	Final loss	Accuracy	AUC-ROC	F-Score	Precision	Recall
**Peptide Triads**								
3 × 2	3,506,094	4,573,534	34,992	0.501	0.502	0.668^3^	0.501	1.000
3 × 3	3,202,563	3,727,467	30,027	0.499	0.499	0.666	0.499	1.000
3 × 4	2,454,016	2,821,820	22,864	0.500	0.499	0.000^4^	0.000	0.000
3 × 5	1,943,073	2,346,432	18,585	0.502	0.500	0.668	0.502	1.000
3 × 7	1,561,153	1,744,130	14,319	0.500	0.500	0.667	0.500	1.000
3 × 9	1,276,502	1,398,539	11,586	0.500	0.500	0.666	0.500	1.000
**Peptide Duos**								
2 × 3	938,992	972,945	8,284	0.501	0.500	0.000	0.000	0.000
2 × 5	692,955	658,592	5,857	0.498	0.500	0.665	0.498	1.000
2 × 7	526,614	506,836	3,625	0.503	0.504	0.523	0.503	0.544
2 × 9	419,945	420,781	2,519	0.501	0.500	0.505	0.500	0.510

Loss function is optimized using the ADAM optimizer and a mini-batch size of 128. ^1^Number of binding peptide fragments samples, and ^2^number of non-binding fragments samples. Calculated probability in the binding output node for all test data set with randomized labels is constant and slightly above 0.5^3^ or below 0.5^4^.

## Discussion

The up to 93% of accuracy (Table 1) and AUC-ROC of 0.979 (Fig. 4) from multi-millions of PT and PD samples shows that intra-protein binding peptide fragments do have specific and intrinsic sequence patterns, which are distinct from the non-binding ones. The learned patterns, encoded in the neural network model, are unlikely computational artifacts. First, no models could be learned from negative control or PTs and PDs of randomized labels. Second, substantial changes in the neural network structure, including number of hidden layers and nodes, and training parameters do not significantly affect the classification performance.

**Figure 5:**
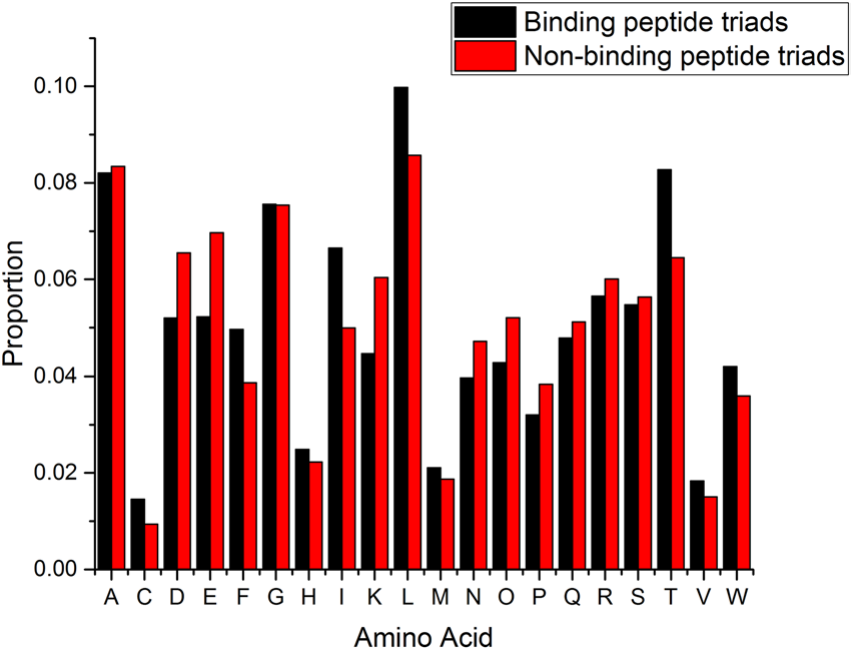
Amino acid composition of binding and non-binding peptide triads of 7 residues. A student t test gives a pvalue of 1.0.

The difference in amino acid composition between BPTs/BPDs and NBPTs/NBPDs is overall insignificant (Fig. 5, pvalue = 1.0). However, hydrophobic residues (I, L, F) seem to be more prevalent in the binding peptide fragments and charged ones (D, E, K) in the non-binding ones (Fig. 5), and one may ask whether the binding is driven by hydrophobic interactions and non-specific. To answer this question, we generated 1,561,153 randomly swapped PTs from 1,561,153 binding PTs of 7 residues long (see Method section for description of the procedure). The binding PTs and the randomly swapped PTs have exactly the same composition of peptide fragments, and the difference is only in the combination. Again, 80% of the combined 3,122,306 samples were used as training set, 10% as validation set, and 10% as testing set. We performed classification of the binding PTs and the randomly swapped PTs using long short-term memory^18^ (LSTM) model of recurrent neural network^19^ (RNN). RNN has demonstrated excellent performance in identify patterns in sequence data such as natural language. For the training, we used 1024 hidden variables, a regularization coefficient of 0.0001, and a learning rate of 0.001, and a mini batch size of 128. We also used the cross entropy as the cost function. The training stopped after 50 iterations when the cost started to rise.

The prediction accuracy on the test data set is 85%, the AUC-ROC is 0.92, the precision is 0.81, and the recall is 0.90. The excellent prediction accuracy provides convincing evidence that the binding between peptide fragments in BPTs is specific and it mainly depends upon correct combination of peptide fragments. Non-specific hydrophobic interactions cannot be fully ruled out, but its role is secondary.

PTs of 7 residues have the best accuracy of 0.931 and AUC-ROC of 0.979. Computationally, if peptide fragments are too short, the neural network model may not have sufficient capacity for the sequence patterns. This is likely true for PTs of 3 residues. On the other side, if the peptide fragments are too long, there may not be sufficient number of samples for training. In this study, 7 seems to be a well-balanced choice. Biochemically, to achieve a binding of sufficient strength that withstand thermal noise, peptide fragments also need to be of sufficient length.

PTs have a significantly better performance than PDs. The best accuracy for PDs is 0.841, lower than the best one for PTs (0.931). This difference is unlikely due to size of the input layer of the neural network. We achieved accuracy of 0.80 with 6.9 millions of PTs of three residues and the input layer size of 180 (3 × 3 × 20). In contrast, for 0.8 million samples of PDs of 9 residues with the input layer size of 360 (2 × 9 × 20), the accuracy is 0.770. As discussed above, RNN model with 1024 hidden variables is capable of differentiating over 1.5 millions of binding PTs from the randomly swapped ones. However, the same model is incapable of classifying binding PDs from the randomly swapped ones. One explanation is that the RNN model is not powerful enough; however, this does not seem likely as intuitively PTs are more complex than PDs. We speculate that binding PTs might be a new natural phenomenon; furthermore, three peptide fragments could geometrically form more compact and stable structure.

In this study, all BPTs and BPDs from known protein X-ray structures are predicted with accuracies of up to 93% and 84%, respectively, and they are apparently helpful in predicting topology and large-scale structure of proteins from amino acid sequence^20^. BPTs plus BPDs are likely important force in forming large scale structures of proteins, and they may provide another explanation to the Levinthal’s paradox^21^. For a protein of 150 residues, assuming a minimum amino acid separation of 10 between two binding fragments, we have roughly 15 chunks. The possible combinations of choosing 3 out of 15 is 455. Thus, a protein, itself a computing machine, may not need to search through astronomical number of possible conformations to find global free energy minimum.

Our finding suggests a hybrid model for protein folding: folding starts with a hydrophobic collapse, followed by formation of BPTs, equivalent of nucleation, and finally acquisition of correct packing interactions. The mechanism of coupled folding and binding^22^ is to some degree relevant to this hybrid hypothesis. Many eukaryotic proteins are disordered under physiological conditions, and fold to ordered structures only on binding to external cellular targets^23^. In the hybrid model, we proposed intra-protein, specific binding between peptide fragments in BPTs as a key step in protein folding process. The mechanism of coupled folding and binding suggests that inter-protein, specific binding may play a similar role.

This research is apparently at a very early stage, but the results, after further improvements and testing, could be applicable for protein structure computation. Given a new protein with known amino acid sequences, binding points could be predicted using the trained neural network models to form a scaffold for the protein. Protein-protein interactions are more complex. But for those involving interactions between consecutive peptides, the deep learning method may be applicable if sufficient number of samples is available and the rules governing inter-protein peptide binding are comparable to those governing intra-protein peptide binding.

**Figure 6:**
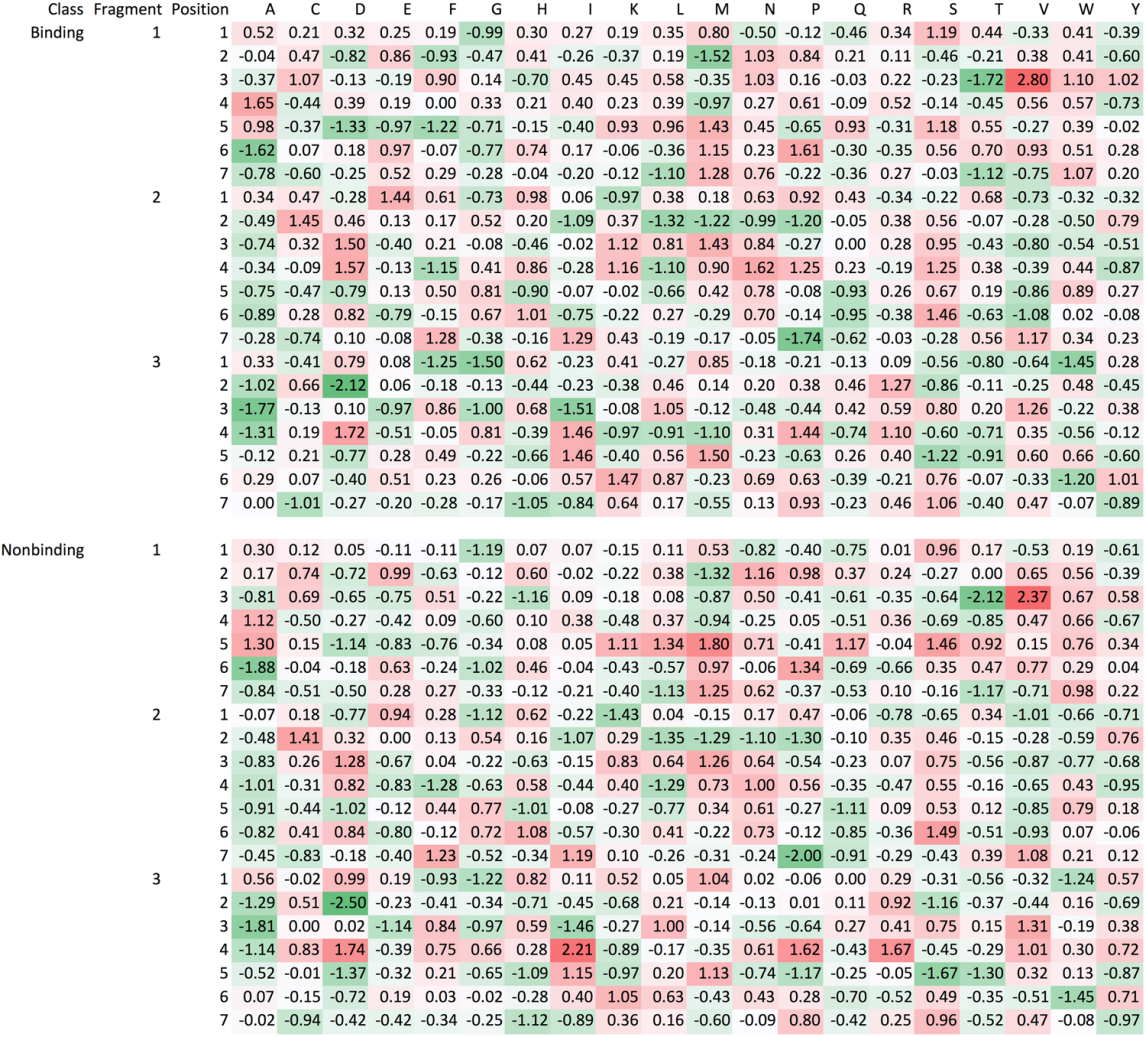
Connection weight from linear model assuming binding as linear sum of contributions from all amino acids. For peptide triad of seven amino acids, there are a total of 21 amino acids (3 × 7) and 420 nodes (3 × 7 × 20) in the input layer. Each amino acid is represented by 20 binary nodes. For example, alanine is represented by 10000000000000000000, and tyrosine by 00000000000000000001. Each node has two connections to the binding node and the non-binding nodes in the output layer, respectively. For an amino acid, the connection weight shown here is the sum over the 20 nodes connecting to the binding and the non-binding nodes; it roughly represents the contribution of the amino acid to the binding and non-binding of peptide triads. A positive value means favoring binding, and a negative value means opposing binding.

It would be very nice if meaningful, and human understandable sequence patterns could be extracted from the trained neural network models. Unfortunately, the model used in this study consists of four hidden layers and each layer consists of 256 neurons; at present, no effective methods are available for this task. In order to extract patterns that are comparable to well-known ones, we trained a linear model, without any hidden layers, over the training data set for PT. In this model, the binding is considered as the result of linear combination of all input variables or amino acids in the peptide fragments, and the trained weights would represent contribution of each amino acid at a given position. The training process converged very quickly in one iteration, which consists of roughly 20,000 minimizations for PTs of 7 residues, and the trained linear model achieved an accuracy and ROC of 0.668 and 0.674, respectively. This linear model is weak, and the heat maps (Fig. 6) for both the binding and non-binding connection weights do not show any obvious patterns. Hydrophobic and charged amino acids do not have significantly enhanced connection weights for binding and non-binding, respectively. This suggests that the relationship between binding and non-binding peptide fragments is mainly nonlinear (in other words, the binding is not proportional to the sum of individual contributions from residues) and unlikely as simple as A-T and C-G in DNA double helix model.

Machine learning algorithms have been applied into prediction of protein contact map with various degree of success^24^. These efforts are based upon the assumption that two residues of a protein are brought together and in contact after the protein’s 3D structure is formed; thus, they use entire protein sequences in the machine learning algorithm. This study is based upon the hypothesis that specific binding between short peptides are the driving force. The excellent performance of the trained neural network supports this hypothesis, and apparently it also benefits from the much larger data sets for training and testing. Many efforts have been made to explore the relationship between point mutation and protein stability^25–28^. The neural network model we proposed in this study is very different from these efforts. On the one hand, the neural network model is capable of capturing complex and non-linear relationships between input peptide fragments and their binding. Similar neural network model could become a powerful tool in studying other complex relationships such as the one between multiple gene mutation and cancer. On the other hand, the resultant model is difficult to decipher. New algorithms and user interfaces are apparently needed to extract human understandable patterns in the trained deep neural network.

## Methods

### Protein structure data

We used 12,946 protein X-ray structures from Protein Data Bank (PDB)^15^ to extract intra-protein peptide fragments, either binding or non-binding. These proteins are from the precompiled culled PDB list^29^, and the goal of the list is to create a non-redundant coverage for all available protein structures. Proteins in this list have an amino acid percent identify <50%, a resolution better than 2.0 A, and a R-factor smaller than 2.5.

### Extraction of peptide triads and peptide duos

An intra-protein BPT is defined as three peptide fragments of a protein having all three pair-wise minimum distances between center residues smaller than 5.0 Å (Figs S1 and S2). For fragments having even number of residues, the minimum distance between two fragments is calculated as the average of minimum distances between two center residues. The minimum distance between two residues is defined as the minimum distance between all non-hydrogen atoms of the two residues. A NBPT is defined as three peptide fragments having all three pair-wise distances between center residues greater than 30 Å. Choosing 30 Å is to produce a balanced training data set, and smaller cutoffs do not affect the training results. To avoid redundancy, if the positions on the amino acid sequences of all three fragments of two PTs, either binding or non-binding, are less than 9 residues away from each other, these two PTs are considered as the same, and only one PT is used. Duplicated PTs (about 5–10%) were eliminated, and the numbers of unique BPTs and NBPTs of 2–9 residues, extracted from 12,946 protein database entries, are given in Table 1.

An intra-protein BPD is similarly defined as BPT, and the difference is in the number of fragments (three vs two) (Figs S1 and S2). One BPT essentially consists of three BPDs. To learn the model for two peptide fragments only, BPDs from BPTs are excluded in the training and test of PDs. We also performed training and testing with BPDs including BPTs, and observed no significant differences.

To perform the deep neural network training, each amino acid is encoded by 20 bit vector or 20 neurons. For PT of seven residues, for example, the total size of the input vector or number of neurons in the input layer is 3 × 7 × 20 = 480. Among the 480 bits or neurons, only 21 (3 × 7) have 1 s, and all rest 0 s.

### Deep learning

Deep Learning^14^ methods, as representation learning methods, allow deep neural networks discovering the representations from raw data for specific tasks such as classification and detection. Supervised learning is the most common form of machine learning which deep learning improves the state-of-the-art of most supervised learning problems. With the help of the ground truth or label of data set, deep learning can learn better representation to predict such ground truth. A loss function captures the distance between the current output of the neural network and the ground truth, then the network propagates the error backwards to adjust all the parameters (weights) in the neural network. In this way, the loss or distance can be significantly reduced after the training process. The binding and non-binding peptide fragments classification is supervised learning with the ground truth as if the peptide fragments are binding or non-binding. Thus, we use deep learning to learn better features and get better classification performance.

We designed a fully connected feedforward neural network of one input layer, four hidden layers, and one output layer for binding and non-binding classification (Fig. 1). For PTs of 2, 3, 4, 5, 7, and 9 residues, the input layer consists of 120, 180, 240, 300, 480, and 540 nodes, respectively. Each hidden layer consists of 256 nodes or neurons. In each hidden layer, the fully-connected layer is followed by the activation function of Rectified Linear Units (ReLU)^30^ which can introduce nonlinearity into the presentation learning. After the hidden layers, Softmax layer is used as the classification layer (or the output layer of two nodes for binding or non-binding). Significant changes in the neural network, including number of hidden layers and nodes, will not significantly affect the classification performance, and 4 hidden layers of 256 nodes tend to produce good results. Backpropagation is used for training the network^31^.

The input to the *j*th node of a hidden layer is calculated according to following equation, where *w*_*i,j*_ is the weight connecting *i*th node of previous layer and *θ*_j_ is the bias.

1 $${X}_{j}=\sum _{i}{w}_{i,j}+{\theta }_{j}$$

All hidden layers use the Rectified Linear Unit as the activation function, and output layer uses Softmax function as the activation function.

We used “cross-entropy” with L2 regularization as the loss function according to the following equation:

2 $$H=\sum _{i}\sum _{j}{y}_{j}^{^{\prime} }\,\mathrm{log}({y}_{j})+\lambda \sum _{i}({w}_{i}^{2}+{\theta }_{i}^{2})$$

where *i* denotes *i* th training sample, *j j* th class, *y* is the predicted probability distribution, *y′* is the true distribution (the one-hot representation of the label), and *λ* is the coefficient for L2 regularization.

Optimization of the loss function is carried out by mini-batch of a size 128 and the ADAM optimizer^16^, which is implemented as tf.train. AdamOptimizer in the Tensorflow library (www.tensorflow.org). The regularization coefficient and starting learning rate were optimized after a grid search (Table 2).

The neural network training and prediction were performed on CyberpowerPC SLC2400C desktop with Intel core i7 and 8GB Nvidia GeForce GTX 1080 graphic processing unit, installed with Ubutun distribution of 16.10, python 3.4, CUDA driver version 8.0, cuDANN version 5.1, and Tensorflow 0.11rc. The python program was written to implement the neural network model (Fig. 1) and optimize the loss function.

BPTs/BPDs and NBPTs/NBPDs were randomly split into three data sets: 80% for training, 10% for validation, and 10% for test (Table 1). The training process was constantly monitored by checking the accuracy of the validation data set, and it was terminated in about 3000 iterations and about 20 hours when either no further improvement was observed or the improvement was deemed too slow to be meaningful. The trained models were applied to the test data set for benchmarking.

For negative control, the label of each PT and PD was randomly assigned as binding (1) or non-binding (0), and the same training procedure and benchmarking were performed.

### Training process

The loss of training data set for peptide triads of 7 residues is plotted versus iterations in Fig. 2. The plots for other training data sets are very similar. For peptide triads of 7 residues, after a dramatic drop in the first 10 iterations, the loss keeps decreasing, but at a significantly reduced speed. This observation is typical of neural network training process, and it also indicates that the hyper parameters have been well optimized. We stopped the training process after about 3000 iterations. It is interesting to see a relatively quick reduction in loss function between iteration 128 and 256. For peptide triads with random labels, no noticeable reduction is observed after first 10 iterations.

The prediction accuracy of validation data set shows similar profiles (Fig. 3). For peptide triads of 7 residues, the accuracy has a fast increase in the first 10 iterations. Afterward, it keeps increasing, but at a much reduced speed. Corresponding to fast decrease in loss function between iteration 128 and 256, we also see a relatively quick increase in accuracy. For peptide triads of randomized labels, the accuracy stays at 0.5 throughout the training process.

### Random swapping of peptide triads

To illustrate the process, let us assume three peptide triads A1-B1-C1, A2-B2-C2, and A3-B3-C3 and list them in a tabular form:

A1 B1 C1

A2 B2 C2

A3 B3 C3

We randomly shuffle each column three times, the number of rows. The above three peptide triads could become:

A3 B1 C2

A1 B3 C1

A2 B2 C3

and then take each row to generate the randomly swapped peptide triads of A3-B1-C2, A1-B3-C1, and A2-B2-C3.

### Amino acid composition

We also compared amino acid composition difference between binding and non-binding peptide triads and observed no difference (pvalue = 1.0, Fig. 5).

## Electronic supplementary material

Supplementary Information
