# Supplementary material for: Specific and intrinsic sequence patterns extracted by deep learning from intra-protein binding and non-binding peptide fragments
Source: Sci Rep. 2017 Nov 2;7:14916. doi: 10.1038/s41598-017-14877-w (PMC5668431; doi:10.1038/s41598-017-14877-w)
Supplement: Supplementary file 1 — Supplementary Information [file 41598_2017_14877_MOESM1_ESM.pdf]

Title: Specific and intrinsic sequence patterns extracted by deep learning from intra-protein binding and non-binding peptide fragments

Authors: Yuhong Wang*[1], Junzhou Huang[2], Wei Li[3], Sheng Wang[2], Chuanfan Ding [1]

Author Affiliations:

[1] Department of chemistry and Laser Chemistry Institute, Fudan University, Shanghai 200433, P. R. China

[2] Department of Computer Science and Engineering, The University of Texas at Arlington, Arlington, TX 76019, USA

[3] School of life science, Jilin University, Changchun 130012, P. R. China

Figure S1. Illustration of binding peptide triad (BPT, green) and binding peptide duo (BPD, brown) in the X-ray crystal structure of 1KPG.

Figure S2. Illustration of binding peptide triad (BPT, green) and binding peptide duo (BPD, brown) in the X-ray crystal structure of 7ODC.

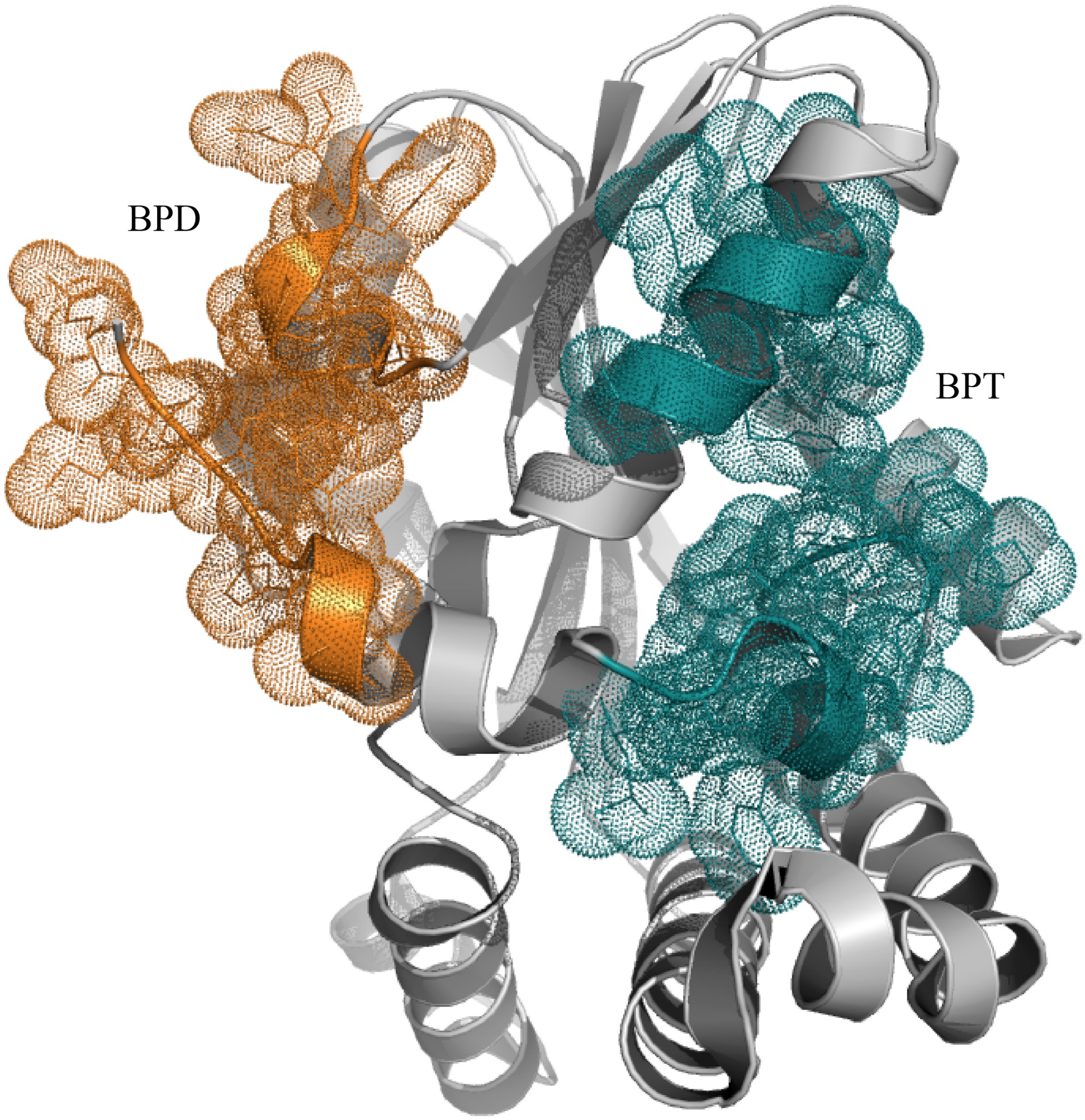
BPD
BPT

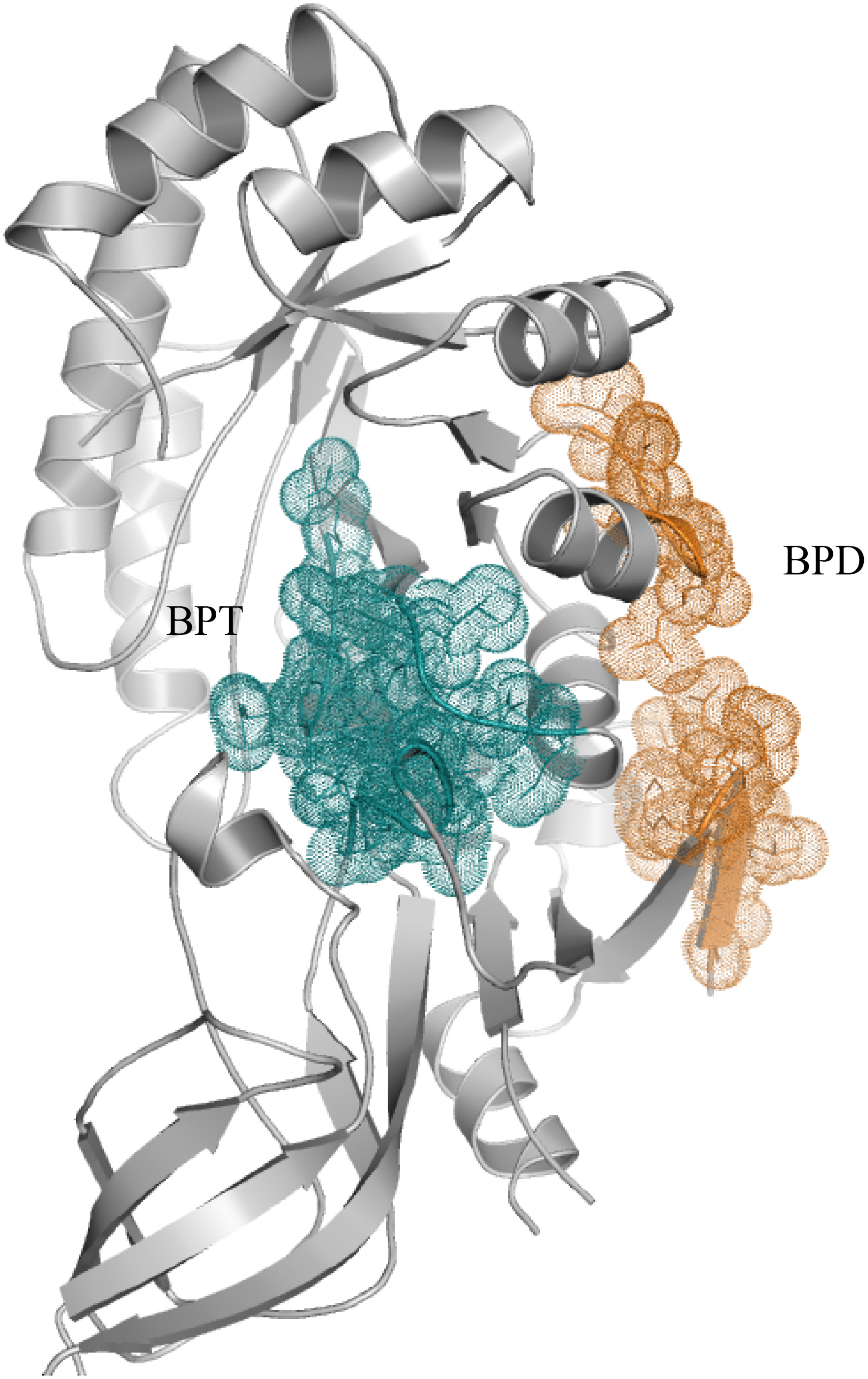
BPT
BPD
